# Cumulative stress, PTSD, and emotion dysregulation during pregnancy and epigenetic age acceleration in Hispanic mothers and their newborn infants

**DOI:** 10.1080/15592294.2023.2231722

**Published:** 2023-07-11

**Authors:** Seyma Katrinli, Alicia K Smith, Stacy S. Drury, Jonathan Covault, Julian D. Ford, Vijender Singh, Bo Reese, Amy Johnson, Victoria Scranton, Pamela Fall, Margaret Briggs-Gowan, Damion J Grasso

**Affiliations:** a Department of Gynecology and Obstetrics, Emory University School of Medicine, Atlanta, GA, USA; b Department of Psychiatry & Behavioral Sciences, Emory University School of Medicine, Atlanta, GA, USA; c Department of Psychiatry and Behavioral Sciences, Boston Children's Hospital, Harvard Medical School, Boston, MA, USA; d Department of Psychiatry, University of Connecticut School of Medicine, Farmington, CT, USA; e Institute for Systems Genomics, University of Connecticut, Storrs, CT, USA; f Computational Biology Core, University of Connecticut, School of Medicine, Storrs, CT, USA; g Center for Genome Innovation, University of Connecticut, Storrs, CT, USA; h Obstetrics & Gynecology, Hartford Hospital, Hartford, CT, USA; i Clinical Research Center Core Laboratory, University of Connecticut School of Medicine, Farmington, CT, USA

**Keywords:** PTSD, GrimAge, gestational epigenetic age, DNA methylation

## Abstract

Pregnancy can exacerbate or prompt the onset of stress-related disorders, such as post-traumatic stress disorder (PTSD). PTSD is associated with heightened stress responsivity and emotional dysregulation, as well as increased risk of chronic disorders and mortality. Further, maternal PTSD is associated with gestational epigenetic age acceleration in newborns, implicating the prenatal period as a developmental time period for the transmission of effects across generations. Here, we evaluated the associations between PTSD symptoms, maternal epigenetic age acceleration, and infant gestational epigenetic age acceleration in 89 maternal-neonatal dyads. Trauma-related experiences and PTSD symptoms in mothers were assessed during the third trimester of pregnancy. The MethylationEPIC array was used to generate DNA methylation data from maternal and neonatal saliva samples collected within 24 h of infant birth. Maternal epigenetic age acceleration was calculated using Horvath’s multi-tissue clock, PhenoAge and GrimAge. Gestational epigenetic age was estimated using the Haftorn clock. Maternal cumulative past-year stress (GrimAge: *p* = 3.23e-04, PhenoAge: *p* = 9.92e-03), PTSD symptoms (GrimAge: *p* = 0.019), and difficulties in emotion regulation (GrimAge: *p* = 0.028) were associated with accelerated epigenetic age in mothers. Maternal PTSD symptoms were associated with lower gestational epigenetic age acceleration in neonates (*p* = 0.032). Overall, our results suggest that maternal cumulative past-year stress exposure and trauma-related symptoms may increase the risk for age-related problems in mothers and developmental problems in their newborns.

## Introduction

Pregnancy can be a stressful period for women due to potential changes in relationships, health-related concerns, preparation for parenthood, and increased financial strain [1,2]. These pregnancy-related stressors coupled with the effects of fluctuating hormones may exacerbate or prompt the onset of psychological impairment, particularly stress-related disorders, such as post-traumatic stress disorder (PTSD) [3–5]. PTSD also may occur as a sequela of pregnancy-related stressors that are traumatic in nature (e.g., intimate partner violence), and it may be precipitated or exacerbated by an accumulation of exposures to stressors that occur during pregnancy but are neither traumatic nor directly related to pregnancy [4]. PTSD is associated with heightened emotional and physiological reactivity and altered regulation of both physiological and behavioural responses to stressors, in addition to increased risk of chronic age-related disorders and earlier mortality [6–8]. Prenatal PTSD prevalence may reach to 40% in low-income, minority populations with known health disparities [8,9]. McEwen’s stress model describes the perinatal period as a ‘window of opportunity’ [10], where maternal stress exposure and PTSD may influence foetal health trajectories via placental-foetal stress physiology [11,12], epigenetic processes [13], and problems with caregiving [14]. Indeed, studies have linked maternal PTSD to negative perinatal outcomes, including pre-term birth and low birth weight [9], as well as developmental problems in young children [15–17].

In addition, lifetime stress exposure [18] and PTSD [19] have been shown to be associated with problems in maternal emotion dysregulation. When mothers of infants or young children experience difficulties in emotion regulation, this has been found to be associated with problems in parenting [20], maternal-child attachment bonding [21], and their child’s development and psychological functioning, such as child social-emotional/behavioural problems [22]. Difficulties in maternal emotion regulation during pregnancy or soon after childbirth may impair maternal-infant emotional co-regulation and precipitate a cascade of problems with parenting, attachment, and infant development and functioning [23,24].

Intergenerational effects of maternal stress exposure and PTSD are also associated with DNA methylation (DNAm) age acceleration in the offspring [25]. DNAm age is a reliable measure of biological age [26–29], which refers to the biological wellbeing of the body based on an individual’s physiological state and cellular health. A significant body of literature supports an association between PTSD and DNAm age acceleration (i.e., higher DNAm age values compared to chronological age) in adults [30–32], measured by Horvath’s multi-tissue clock [26] and Hannum’s blood-specific clock [27]. These earlier epigenetic clocks, Horvath’s multi-tissue clock [26] and Hannum’s blood-specific clock [27], were trained on chronological age and proposed as a measure of biological ageing. However, the second-generation epigenetic clocks, PhenoAge that includes clinical biomarkers as predictors [28], and GrimAge that was developed by regressing time-to-death on DNAm based age-related biomarkers and smoking packs per year [29], were proposed as an epigenetic predictors of healthspan and long-term mortality risk. Recent evidence reports accelerated DNAm GrimAge in those with PTSD [33–37]. Of those, the study of Katrinli et al. conducted in a predominantly African American cohort showed that individuals with current and lifetime PTSD have accelerated GrimAge, which also associated with cortical atrophy in brain areas related to emotion processing and threat response [33]. Yang et al. reported accelerated GrimAge in male veterans with PTSD, as well as a significant association between longitudinal changes in GrimAge acceleration and PTSD symptoms at 3-years follow-up [34].

However, the measures of DNAm age acceleration do not accurately translate to earlier phases of life in which deceleration rather than acceleration of ageing represents a potential adverse outcome. Hence, recent efforts have focused on developing gestational epigenetic clocks predictive of gestational age (i.e., gestational epigenetic age) [38–42]. The prediction accuracy of gestational epigenetic age clocks is more concordant with gestational age predictions assessed with the gold standard of ultrasound than with predictions based on the last menstrual period (LMP) [39]. The difference between gestational epigenetic age and clinically estimated gestational age (i.e., gestational epigenetic age acceleration) may serve as a surrogate indicator of the physiological development of the neonate, with higher gestational epigenetic age acceleration values (i.e., accelerated gestational epigenetic age) potentially indicating more developmental maturity in neonates [39]. Recently, Suarez et al. reported a significant association between maternal depression and lower gestational epigenetic age acceleration, which in turn is associated with developmental problems in boys [43]. A recent study by Koen and colleagues, conducted on 271 Southern African mothers and infant dyads, showed that maternal PTSD was associated with lower gestational epigenetic age acceleration in offspring [44]. Cumulative exposure to recent stressors is associated with both depression [45] and PTSD [46]; but, the impact of maternal cumulative stress exposure, PTSD, and premature epigenetic ageing on the gestational epigenetic age acceleration of their newborn child has not been investigated.

Therefore, we first aimed to evaluate the association between past-year cumulative stress exposure, prenatal PTSD symptoms, and premature accelerated ageing in women, using three different epigenetic clocks, Horvath’s multi-tissue clock, PhenoAge, and GrimAge. Then, we sought to investigate the relationship of maternal stress exposure and PTSD symptoms with gestational epigenetic age acceleration in their newborn child.

## Materials and methods

### Sample

**Figure 1.**
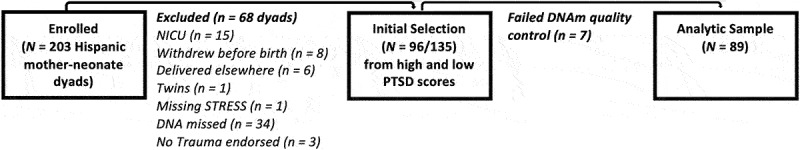
Overview of analytic sample.

Salivary DNA samples from 96 mother-neonatal dyads were selected for a genome-wide methylation analysis from a cohort of 203 Hispanic/Latina participants enrolled in a larger study focused on maternal trauma and prenatal stress. The selection was made to test research aims from two supplemental projects [information withheld] budgeted to assay samples from 96 dyads using the MethylationEPIC BeadChip (Illumina). Among the cohort of 203 women, 68 were excluded from selection due to exclusion criteria of the larger study (i.e., infant placed in the NICU, twins, delivered elsewhere precluding collection of samples at birth), exclusion criteria of the current study (i.e., no trauma endorsed on the trauma history questionnaire), study withdrawal prior to sample collection, or missing key data (i.e., missing maternal and/or neonatal DNA sample, full trauma history and symptom data). Among the 135 remaining dyads, 96 were selected such that 48 were drawn sequentially starting from the highest maternal PTSD symptom score and moving downwards and 48 were drawn sequentially starting from the lowest PTSD symptom score and moving upwards. This approach was to ensure sufficient variability in PTSD symptoms. Among the 96 dyads selected for the current study, 7 dyads were removed following DNA methylation quality control (described below), resulting in an analytic sample of 89 dyads. See Figure 1 for a flowchart of the analytic sample.

### Procedure

Third-trimester patients seen at an urban prenatal care clinic were invited to participate. Eligibility criteria included English-speaking, singleton pregnancy, and plan to deliver at [information withheld] Hospital. Participants provided written, informed consent in private locations and were asked to summarize their understanding of material before enrolment. Enrolled participants completed self-report measures of past-year stressful events, lifetime trauma history, PTSD symptoms, and emotion dysregulation. Salivary DNA samples were collected from mothers and infants within 24 h of delivery. All included study mothers reported at least one type of past exposure to traumatic event(s), and all infants who had a gestational age of at least 34 weeks. All study procedures and protocols were approved by institutional review boards from the [information withheld].

### Measures

Demographic information on age, race, and smoking (lifetime and during pregnancy) was collected by self-report. Newborn characteristics (i.e., sex, gestational age, head circumference, length, weight) were obtained via medical chart extraction. Past-year stressful life events (e.g., serious accident or injury, family went on welfare) were assessed using the widely used and validated Turner life events scale (LES) [47]. LES score was operationalized as a continuous measure, by summing 34 dichotomous items assessing the presence of past-year events.

Lifetime trauma history and DSM-5 PTSD symptoms were assessed via self-report with the Structured Trauma-Related Experiences and Symptoms Screener for Adults (STRESS-A) [8,48]. Part I assessed lifetime exposure to 29 PTSD qualifying traumatic events. In Part II, respondents rated the frequency of symptoms over the past week on a 4-point rating scale (0=*none*, 1=*1 day*, 2=*2–3 days*, 3=*most days*). Cronbach’s *α* for total symptom severity was 0.949.

Emotion dysregulation was assessed with the Difficulties in Emotion Regulation Scale (DERS) [49]. The DERS is a 36-item self-report measure of six dimensions of emotion dysregulation: Non-acceptance, Goals, Impulse, Strategy, Clarity, and Awareness. Respondents indicate how often each statement applies to them on a 5-point rating scale (1=*almost never*, 2=*sometimes*, 3=*about half the time*, 4=*most of the time*, 5=*almost always*). The DERS exhibits good reliability and validity among racially diverse demographic groups. The DERS total score (sum of subscales) was used (α= .927).

### DNA methylation and epigenetic clocks

Saliva samples were collected from mothers using Oragene DNA collection kits. Saliva samples from neonates were collected using five micro-sponges. Both samples were collected within 24 hours post-delivery. DNA was extracted from saliva samples using the Oragene Prepit L2P procedure by DNA Genotek and assayed using the MethylationEPIC BeadChip (Illumina). The R package *CpGassoc* was used for quality control steps, including i) removal of samples with probe detection call rates < 90% and an average intensity value of either < 50% of the experiment-wide sample mean or < 2000 arbitrary units (removed 4 mother-neonatal dyad samples and 3 neonatal samples); ii) setting probes with detection p-values >0.01 as missing; and iii) filtering out missing probes for > 10% of samples [50]. Probes that were known to cross-hybridize between autosomes and sex chromosomes were filtered out [51]. Methylation data was preprocessed and normalized using single-sample Noob (ssNoob) background correction implemented in R package *minfi* [52]. Batch effects of chip and position were removed using ComBat [53]. We also excluded mother pairs of 3 failed neonatal samples. The analytical sample included 89 mother-neonatal dyads.

DNAm data was used to estimate saliva cellular composition, and a smoking score for each sample. Saliva cell proportions (i.e., epithelial, fibroblast, CD8+T, CD4+T, natural killer (NK), B cells, monocytes, and neutrophils) was estimated using the *hepidish* function from R package *Epidish* [54]. The smoking score was generated using the weights of 39 CpG sites associated with smoking pack years [55] (Supplementary Table S1), as previously described [56].

Horvath’s online calculator (https://dnamage.genetics.ucla.edu/new) was used to calculate epigenetic age and age acceleration (age-adjusted epigenetic age) measures, Horvath’s multi-tissue clock, PhenoAge, and GrimAge. Gestational epigenetic age of infants was calculated based on the recent EPIC-based gestational clock developed by Haftorn and colleagues [42]. We regressed gestational epigenetic age on chronological gestational age in weeks to obtain gestational epigenetic age acceleration.

### Statistical analyses

We first evaluated the correlation between epigenetic age estimates and chronological age in mothers, and the correlation between DNAm gestational age and chronological gestational age in infants to validate the accuracy of these epigenetic clocks for saliva samples.

In mothers, we tested the correlations between epigenetic age acceleration measures and potential confounders, including cell proportions, self-reported smoking and marijuana use (lifetime and during pregnancy), and DNAm-derived smoking score. We conducted separate linear regressions to test the associations between each epigenetic age acceleration measures (GrimAge acceleration, PhenoAge acceleration, and Horvath’s DNAmAge acceleration) and each stress and trauma-related measures (i.e., LES, STRESS, DERS). We then employed full models with each epigenetic age acceleration measures as the dependent variables and all three trauma-related measures as independent variables. All models were adjusted for proportions of monocyte, B cell, NK, CD4+T and epithelial cells. Since, GrimAge incorporates DNAm surrogate of smoking assessed by smoking packs per year, a DNAm-based smoking score was not included in the regression model for GrimAge. For PhenoAge and Horvath’s DNAmAge models, post-hoc sensitivity analyses explored the possible confounding effect of smoking by including DNAm-based smoking score as a covariate.

In infants, we tested the correlations of gestational epigenetic age acceleration with cell proportions, anthropomorphic variables of infants (e.g., sex, length, weight, and head circumference), and smoking status of mothers. We used a linear regression model to test the association between gestational epigenetic age acceleration and maternal stress and trauma-related measures (i.e., LES, STRESS, DERS), including infant’s sex and estimated cell proportions (i.e., monocyte, B cell, NK, CD4+T and epithelial cells) as covariates. Since, fibroblast and CD8+T proportions were too low, they were not included as covariates. A post-hoc sensitivity analysis also examined the possible confounding effect of maternal smoking by including maternal DNAm-based smoking score as a covariate.

## Results

### Demographic and clinical characteristics

Demographic and clinical characteristics of mothers and their newborn infants are presented in Table 1. All mothers in this study self-identified as Hispanic. None of the subjects reported alcohol use during pregnancy. Correlations between sociodemographic characteristics and study variables are presented in Supplementary Figure S1.

**Table 1.** Demographic and clinical characteristics of the study.

Characteristics of Mothers (*N* = 89)	Mean (SD), range or N (%)
Age	26.93 (5.09), 18.28–38.10
Race/Ethnicity	
White/Hispanic	31 (34.8%)
Black/Hispanic	7 (7.9%)
Other/Hispanic	51 (57.3%)
Annual income < $20,000	47 (75.8%)^a^
Unemployed	46 (51.7%)
Medicaid/Medicare	79 (95.2%)^b^
Less than high school diploma or GED	26 (29.9%)^c^
Tobacco use during pregnancy	8 (9.0%)
Tobacco use ever	25 (28.1%)
Marijuana use during pregnancy	10 (11.2%)
Marijuana use ever	21 (23.6%)
Smoking score^#^	14.7 (19.81), −22.82–79.34
LES past-year stressful events	3.3 (2.89), 1–13
STRESS PTSD Symptoms	18.37 (15.73), 0–51
DERS Emotion Dysregulation	72.11 (23.39), 36–129
Characteristics of Newborns (*N* = 89)	
Sex (% Female)	55 (61.8%)
Gestational age (weeks)	39.17 (1.21), 34.29–41.43
Head Circumference (Centimeters)	33.94 (1.20), 30.50–36.50
Length (Inches)	19.76 (0.90), 17.50–22.00
Weight (Grams)	3261.73 (421.35), 2150–4120

Note. ^a^Missing for 27 participants. ^b^Missing for 6 participants. ^c^Missing for 2 participants. ^#^The description of 39 CpG sites that were used to calculate the smoking score were presented in Supplementary Table 1.

### Life stress, PTSD symptoms, and emotion regulation problems associate with GrimAge

All three epigenetic age measures (GrimAge, PhenoAge, and Horvath’s DNAmAge) strongly correlated with maternal chronological age (*r* > 0.85, *p* < 2.2e-16, Supplementary Figure S2). Of the three epigenetic clocks, GrimAge showed the strongest correlation with chronological age (*r* = 0.89, *p* < 2.2e-16). Pairwise correlations between epigenetic age acceleration measures and potential confounders, including cell proportions, self-reported smoking and marijuana use (lifetime and during pregnancy), and DNAm-derived smoking score were shown in Supplementary Figure S3.

**Figure 2.**
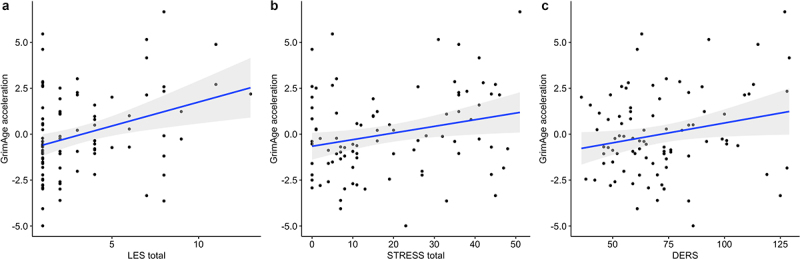
Associations between GrimAge acceleration and (a) number of past-year stressful life events (LES), (b) PTSD symptoms (STRESS), and (c) difficulties in emotion regulation in mothers (DERS). Results are from multiple linear regression models, adjusted for proportions of monocyte, B cell, NK, CD4+T and epithelial cells. Gray shaded area represents 95% confidence region.

Exposure to past-year stressful life events (Figure 2a), PTSD symptoms (Figure 2b), and difficulties in emotion regulation (Figure 2c) were each significantly associated with accelerated GrimAge in mothers (Table 2), suggestive of premature accelerated ageing. Exposure to past-year stressful life events was also associated with accelerated PhenoAge (Table 2). However, the association did not remain significant in the post-hoc sensitivity analysis that adjusts for DNAm-based smoking score (beta estimate = 0.23, *p* = 0.08). Horvath’s DNAmAge acceleration was not associated with any stress and trauma-related measures (Table 2). In the full model that includes all three trauma-related measures, only past-year stressful life events remained associated with GrimAge acceleration and PhenoAge acceleration (Table 2).

**Table 2.** Associations of GrimAge acceleration and gestational epigenetic age acceleration with maternal stress, PTSD symptoms, and difficulties in emotion regulation.

	Beta Estimate	SE	t-value	p-value
*GrimAge acceleration (separate linear regressions)*				
LES past-year stressful events	0.31	0.08	3.76	**3.23e-04**
STRESS PTSD symptoms	0.04	0.02	2.39	**0.019**
DERS emotion dysregulation	0.02	0.01	2.23	**0.028**
*GrimAge acceleration (full model)*				
LES past-year stressful events	0.28	0.11	2.58	**0.012**
STRESS PTSD symptoms	5.89e-04	0.02	0.03	0.98
DERS emotion dysregulation	7.97e-03	0.01	0.65	0.52
*PhenoAge acceleration (separate linear regressions)*				
LES past-year stressful events	0.27	0.12	2.29	**0.025**
STRESS PTSD symptoms	7.89e-03	2.24e-02	0.35	0.73
DERS emotion dysregulation	4.26e-03	1.52e-02	0.28	0.78
*PhenoAge acceleration (full model)*				
LES past-year stressful events	0.41	0.15	2.64	**0.010**
STRESS PTSD symptoms	−3.17e-02	2.91e-02	−1.09	0.28
DERS emotion dysregulation	−7.80e-03	1.76e-02	−0.44	0.66
*Horvath’s multi-tissue age acceleration (separate linear regressions)*				
LES past-year stressful events	9.42e-03	1.10e-01	0.09	0.93
STRESS PTSD symptoms	−7.20e-03	2.00e-02	−0.36	0.72
DERS emotion dysregulation	8.42e-03	1.35e-02	0.62	0.54
*Horvath’s multi-tissue age acceleration (full model)*				
LES past-year stressful events	0.03	0.14	0.19	0.85
STRESS PTSD symptoms	−0.02	0.04	−0.78	0.44
DERS emotion dysregulation	0.01	0.02	0.87	0.39
*Gestational epigenetic age acceleration (separate linear regressions)*				
LES past-year stressful events	−7.78e-03	9.43e-03	−0.83	0.41
STRESS PTSD symptoms	−3.74e-03	1.72e-03	−2.18	**0.032**
DERS emotion dysregulation	−1.87e-03	1.16e-03	−1.61	0.11

Note. Results are from multiple regressions, controlling for sex (only in infants), and proportions of monocyte, B cell, NK, CD4+T and epithelial cells. Significant effects are shown in bold. SE = standard error.

### Gestational epigenetic age acceleration of infants associates with maternal PTSD symptoms

Newborn infants’ gestational epigenetic age was correlated with their chronological gestational age (*r* = 0.38, *p* = 2.9e-04, Supplementary Figure S4). Gestational epigenetic age acceleration was not correlated with infant’s length at birth, weight, or head circumference, but correlated with infant sex, with females having higher gestational epigenetic age acceleration (Supplementary Figure S5). Gestational epigenetic age acceleration was positively correlated with the proportions of epithelial cells, and negatively correlated with proportions of B cells and monocytes. There was no significant correlation between gestational epigenetic age acceleration and smoking status of mothers for both self-reported smoking and DNAm derived smoking score. Gestational epigenetic age acceleration of neonates was correlated with maternal PhenoAge acceleration (*r* = 0.22, *p* = 0.038), but not with maternal GrimAge (*r* = −0.16, *p* = 0.14) and Horvath’s DNAmAge acceleration (*r* = 0.09, *p* = 0.40).

**Figure 3.**
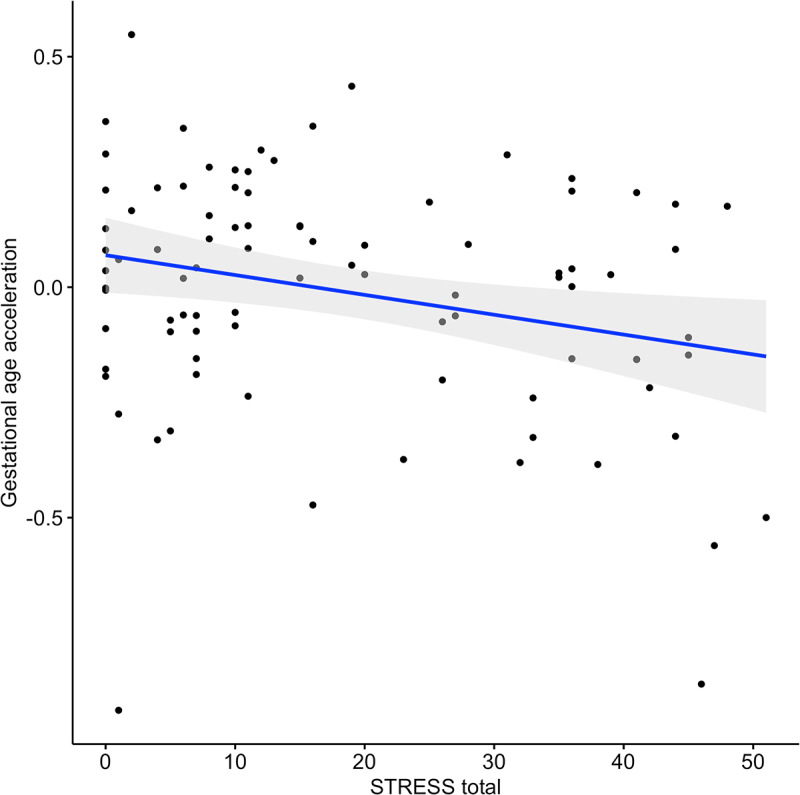
Association between gestational epigenetic age acceleration and maternal PTSD symptoms (STRESS). Results are from multiple linear regression models, adjusted for sex and proportions of monocyte, B cell, NK, CD4+T and epithelial cells. Gray shaded area represents 95% confidence region.

Maternal PTSD symptoms were negatively associated with their newborn’s gestational epigenetic age acceleration (Table 2, Figure 3), suggestive of a plausible delayed foetal development. The association remained significant with the same direction of effect in the sensitivity analysis adjusted for maternal smoking score (beta estimate = −3.58e-04, *p* = 0.49). In the stratified analysis for neonatal sex, maternal PTSD symptoms were negatively associated with gestational epigenetic acceleration in female neonates (beta estimate = −0.005, *p* = 0.019), but not in males (beta estimate = −0.001, *p* = 0.68). However, neonatal sex did not moderate the association between maternal PTSD symptoms and their newborn’s gestational epigenetic age acceleration (beta estimate = −0.002, *p* = 0.57). Infant gestational epigenetic age acceleration did not associate with maternal past-year stressful life events or emotion dysregulation (Table 2).

## Discussion

In this study, we evaluated the associations between stress and trauma-related metrics and epigenetic indicators of ageing and mortality risk in mother-neonatal dyads. Our major findings were as follows: 1) Maternal exposure to past-year stressful life events were associated with accelerated GrimAge and PhenoAge, whereas PTSD symptoms and emotion dysregulation were only associated with accelerated GrimAge; 2) In addition, maternal PTSD symptoms, although not maternal report of stress exposure or difficulty with emotion regulation, were associated with lower gestational epigenetic age acceleration in neonates.

Our findings from saliva were consistent with previous reports from blood linking accelerated GrimAge to trauma burden and PTSD [33–36]. Saliva is composed of a mix of epithelial cells and leukocytes (the main component of blood tissue). Although GrimAge was developed in blood samples, GrimAge estimates calculated from blood and buccal (predominantly consisting of epithelial cells) samples from the same *N* = 21 subjects were shown to be correlated (*r* = 0.48, *p* < 0.001) [57]. In addition, multiple studies report that saliva derived GrimAge were strongly correlated with chronological age (*r* > 0.6, *p* < 0.001) [57–59]. Notably, a recent longitudinal study reported that PTSD symptom severity upon trauma exposure associated with GrimAge acceleration calculated from saliva samples [37]. Since GrimAge was trained on time-to-death and incorporates DNAm-based surrogates of age-related biomarkers, it can accurately predict mortality and morbidity [60]. Hence, accelerated GrimAge in mothers who experienced a greater number of past-year stressful life events and have higher PTSD symptoms is suggestive of a greater risk of earlier mortality. On the other hand, exposure to past-year stressful life events, but not PTSD symptoms or difficulty with emotion regulation, were associated with higher PhenoAge acceleration. These findings were consistent with previous studies that examined all three epigenetic clocks and showed only GrimAge acceleration, but not PhenoAge and Horvath’s DNAm acceleration, was associated with PTSD [35,36].

In our neonate samples, females showed higher gestational epigenetic age acceleration compared to males. Although adult males exhibit higher biological age (i.e., epigenetic age acceleration) compared to females [26], the same phenomenon doesn’t apply to gestational epigenetic age acceleration. Adult epigenetic age acceleration is a predictor of biological ageing [26], whereas gestational age acceleration reflects offspring development and maturity [39]. Hence, there is no well-established sex bias for gestational epigenetic age acceleration, with some studies reporting higher gestational epigenetic age acceleration in female offspring [61–63], and others in male offspring [64–66]. To further evaluate sex differences in gestational epigenetic age acceleration, we conducted a sex-stratified analysis and evaluated neonate sex as a moderator of the association between gestational age acceleration and maternal PTSD symptoms. Our findings did not show a sex-specific association between gestational age acceleration and maternal PTSD symptoms. In addition, gestational epigenetic age acceleration was correlated with maternal PhenoAge acceleration, but not with maternal GrimAge or Horvath’s DNAmAge acceleration. To the best of our knowledge, no study to date reported a correlation between gestational epigenetic age acceleration and maternal epigenetic age acceleration.

Although maternal PTSD symptoms was correlated with both emotional dysregulation (*r* = 0.53, *p* < 0.01), and exposure to past-year stressful life events (*r* = 0.59, *p* < 0.01, Supplementary Figure S1), only maternal PTSD symptoms were associated with gestational epigenetic age deceleration in neonates. Since LES only measures recent stressful and traumatic events, whereas STRESS assesses lifetime events and associated PTSD symptoms, we can speculate that cumulative exposure and trauma-specific symptoms might be necessary for reliable epigenetic changes to occur. In addition, PTSD usually co-occurs with immune dysregulation [67], which might influence gestational epigenetic age acceleration [68].

At first glance, the findings in neonates may seem contradictory to our findings in mothers and previous adult studies that reported associations between accelerated epigenetic age and trauma-related experiences and symptoms [30–34,37]. Moreover, children at ages 6 to 13 years exposed to neighbourhood violence also exhibited higher epigenetic age acceleration [25]. However, epigenetic age acceleration measures trained on adult and paediatric samples are not suitable for use in neonates, nor for estimating gestational age [39]. Since these epigenetic age clocks are not trained on gestational age data, they do not correlate with gestational age [39,69]. Studies investigating the associations of gestational epigenetic age acceleration with prenatal adversities and foetal growth yield mixed results. Lower gestational epigenetic age acceleration has been associated with i) prenatal maternal health adversities, including assisted delivery, prior insulin-treated gestational diabetes mellitus, maternal Sjögren’s syndrome, maternal history of depression, and antenatal depressive symptom severity [43,63,70–72]; ii) prenatal stress assessed using the cerebroplacental ratio [70]; iii) male neonate sex [61–63]; iv) neonatal development delay, including lower birth weight, length, and head circumference, development of bronchopulmonary dysplasia and higher need for respiratory intervention [62,63]; and v) prospective child psychiatric problems [43]. Contradicting reports have shown associations between accelerated gestational epigenetic age and lower birth length, lower 1-min Apgar scores, male neonate sex, maternal preeclampsia, and maternal age greater than 40 years at delivery [41,63–66]. Nevertheless, our results align with a recent study reporting an association between lower gestational epigenetic age and maternal trauma-related experiences and symptoms [44]. The findings of our study and others linking maternal prenatal mental health problems (e.g., maternal antenatal depressive symptoms or PTSD) with lower gestational epigenetic age acceleration in the neonates are aligned with the Developmental Origins of Health and Diseases (DOHaD) hypothesis, which suggests that experiences during the prenatal period can influence the developmental trajectory of the foetus [73].

This study is not without limitations. Firstly, the relatively small sample size restricted the statistical power of our analyses. Even so, we have reported significant associations of GrimAge acceleration with stress and trauma-related metrics and PTSD symptoms, as well as an association between gestational epigenetic age acceleration and maternal PTSD symptoms, which is consistent with previous reports [33–35,37,44].

Second, our cohort is racially homogenous and only includes mother-neonatal dyads of Hispanic ethnicity. Hence, it should be acknowledged that Hispanics exhibit lower GrimAge acceleration compared to white populations, and our results may not generalize to other ethnic or racial groups [74]. However, there is an empiric need to increase research in Hispanic and other minoritized groups, as such our focus on data within Hispanic families has specific relevance. Future studies that examine effects between different minoritized populations, rather than the typical comparison of minority groups to whites, are needed to better understand how structural inequity may differentially effect minoritized groups, a key knowledge gap when considering culturally responsive intervention and prevention efforts.

Third, all participants are from a low socioeconomic group, with 75.8% of participants reporting an annual income < $20,000 and 95.2% relying on Medicare/Medicaid. Since there was no substantive variability in socioeconomic status, the models were not adjusted for socioeconomic status, which has been associated with epigenetic age acceleration [57].

Fourth, we used GrimAge and PhenoAge clocks that were originally developed in blood samples to estimate epigenetic age in saliva samples. However, the chronological age is strongly correlated with both GrimAge (*r* = 0.89, *p* < 2.2e-16) and PhenoAge estimates (*r* = 0.85, *p* < 2.2e-16), indicating that PhenoAge and GrimAge are applicable to saliva samples in our study. Levine and colleagues also demonstrated that PhenoAge shows strong blood-saliva correlation (*r* = 0.81, *p* = 7e-60) [28]. In addition, multiple studies report that saliva-derived GrimAge were strongly correlated with chronological age (*r* > 0.6, *p* < 0.001) [57–59]. Notably, a recent longitudinal study reported that PTSD symptom severity upon trauma exposure is associated with GrimAge acceleration calculated from saliva samples [37].

Fifth, we used the recent EPIC-based gestational epigenetic clock trained on cord blood samples to predict gestational epigenetic age in our neonate saliva samples, as no gestational epigenetic clock specific to saliva tissue yet exists. However, the significant -albeit moderate- correlation between gestational epigenetic age and chronological gestational age (*r* = 0.38, *p* = 2.9e-04), despite being weaker compared to studies that used the Haftorn clock in cord blood (*r* = 0.43–0.65, *p* < 0.001) [61,64,72,75,76], suggests that the recent EPIC-based gestational epigenetic clock is applicable to saliva samples in our study.

Finally, we did not examine or control for the effects of neither maternal and pregnancy health (e.g., gestational diabetes, maternal obesity) nor cumulative stress prior to pregnancy. Hence, we were not able to segregate the impacts of stress during pregnancy on GrimAge acceleration and gestational epigenetic age acceleration from earlier life stress and maternal physical health.

In conclusion, our study shows that pregnant women reporting exposure to more past-year stressors and exhibiting more PTSD symptoms and difficulties with emotion regulation demonstrated accelerated GrimAge soon after childbirth, suggestive of a higher mortality risk. Further, infants born to mothers with higher PTSD symptoms during pregnancy exhibit lower gestational epigenetic age acceleration, which may indicate elevated risk of later developmental problems [43]. Future prospective studies with larger sample sizes are required to investigate possible interactions between PTSD symptoms and GrimAge acceleration in relation to gestational epigenetic age acceleration and prospective child outcomes.

## Supplementary Material

Supplemental Material

## Data Availability

The data that support the findings of this study are available from the corresponding author, DJG, upon reasonable request.
