# Supplementary material for: Cumulative stress, PTSD, and emotion dysregulation during pregnancy and epigenetic age acceleration in Hispanic mothers and their newborn infants
Source: Epigenetics. 2023 Jul 11;18(1):2231722. doi: 10.1080/15592294.2023.2231722 (PMC10337495; doi:10.1080/15592294.2023.2231722)
Supplement: Supplemental Material [file KEPI_A_2231722_SM5956.docx]

**Supplementary Information**

Supplementary Table 1: Description of 39 CpG sites used to calculate the smoking score

| CpG | CHR | Loci | Weights |
| --- | --- | --- | --- |
| cg01692968 | 9 | 9q31.1 | -2.37 |
| cg01940273 | 2 | 2q37.1 | -8.99 |
| cg03329539 | 2 | 2q37.1 | -6.27 |
| cg03636183 | 19 | F2RL3 | -4.65 |
| cg03646329 | 13 | LPAR6 | -2.47 |
| cg03991871 | 5 | AHRR | -5.59 |
| cg04885881 | 1 | 1p36.22 | -2.16 |
| cg05284742 | 14 | ITPK1 | -0.49 |
| cg05575921 | 5 | AHRR | -10.68 |
| cg05951221 | 2 | 2q37.1 | -10.32 |
| cg06126421 | 6 | 6p21.33 | -5.92 |
| cg06226150 | 20 | SLC2A4RG | -1.37 |
| cg07339236 | 20 | ATP9A | -2.71 |
| cg07826859 | 7 | MYO1G | -1.75 |
| cg08709672 | 1 | AVPR1B | -1.05 |
| cg09022230 | 7 | TNRC18 | 0.73 |
| cg09935388 | 1 | GFI1 | -6.87 |
| cg10750182 | 10 | C10orf105 | -0.64 |
| cg11660018 | 11 | PRSS23 | -3.74 |
| cg11902777 | 5 | AHRR | -7.19 |
| cg14580211 | 5 | C5orf62 | -0.43 |
| cg14753356 | 6 | 6p21.33 | -2.64 |
| cg14817490 | 5 | AHRR | -3.31 |
| cg15187398 | 19 | MOBKL2A | -1.28 |
| cg17287155 | 5 | AHRR | -1.67 |
| cg19572487 | 17 | RARA | -4.19 |
| cg19859270 | 3 | GPR15 | -2.69 |
| cg21161138 | 5 | AHRR | -4.3 |
| cg21611682 | 11 | LRP5 | -2.04 |
| cg21733098 | 12 | 12q24.32 | -1.78 |
| cg23161492 | 15 | ANPEP | -5.41 |
| cg23916896 | 5 | AHRR | -4.32 |
| cg23931381 | 19 | ARRDC2 | 0.71 |
| cg24859433 | 6 | 6p21.33 | -1.45 |
| cg25189904 | 1 | GNG12 | -7.02 |
| cg25648203 | 5 | AHRR | -2.25 |
| cg25949550 | 7 | CNTNAP2 | -2.8 |
| cg26271591 | 2 | NFE2L2 | -0.44 |
| cg26703534 | 5 | AHRR | 0.28 |

**
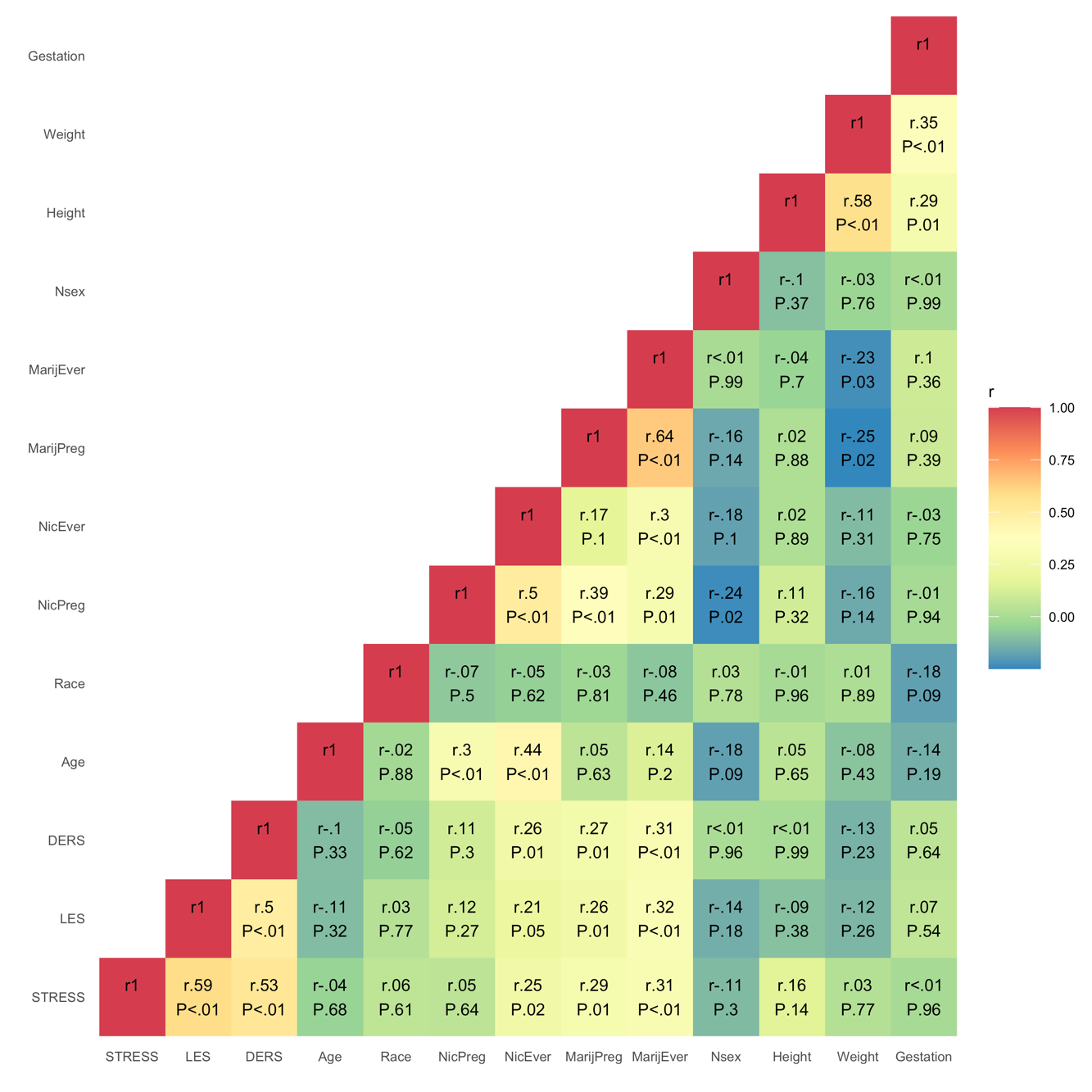
**

Supplementary Figure 1. Correlation heatmap of demographics and study variables. The deeper color indicates stronger correlations. STRESS: Structured Trauma-Related Experiences and Symptoms Screener for Adults. LES: Turner life events scale. DERS: Difficulties in Emotion Regulation Scale. NicPreg: Nicotine use during pregnancy. NicEver: Lifetime nicotine use. MarjPreg: Marijuana use during pregnancy. MarjEver: Lifetime marijuana use. Nsex: Neonate sex.

**
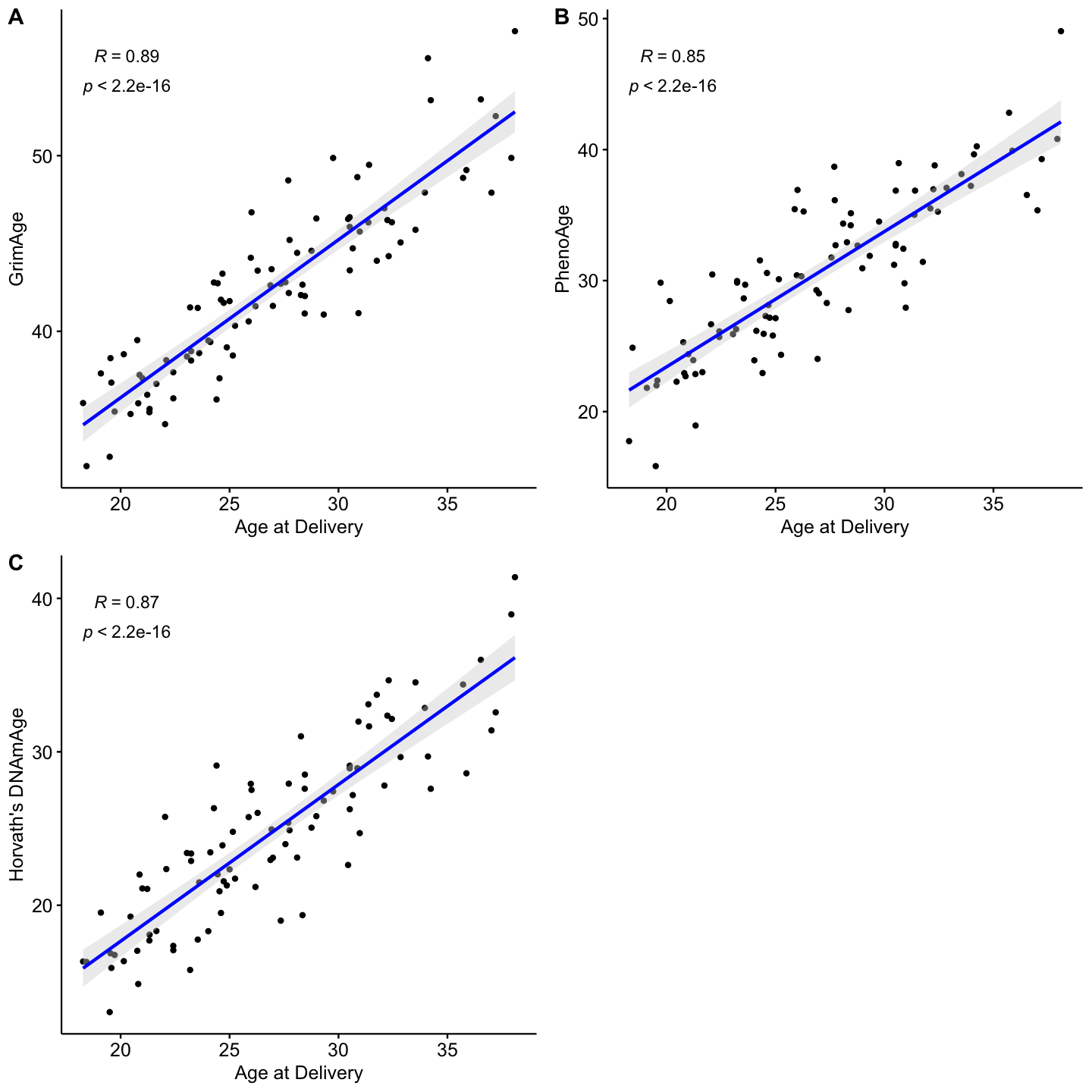
**

Supplementary Figure 2. Correlations between chronological age and DNAm age estimates. A) GrimAge, B) PhenoAge, and C) Horvath’s DNAmAge are correlated with chronological age of mothers at delivery. X-axis shows chronological age (in years) at delivery. Y-axis shows DNAm age estimates (in years). Gray shaded area represents 95% confidence region.


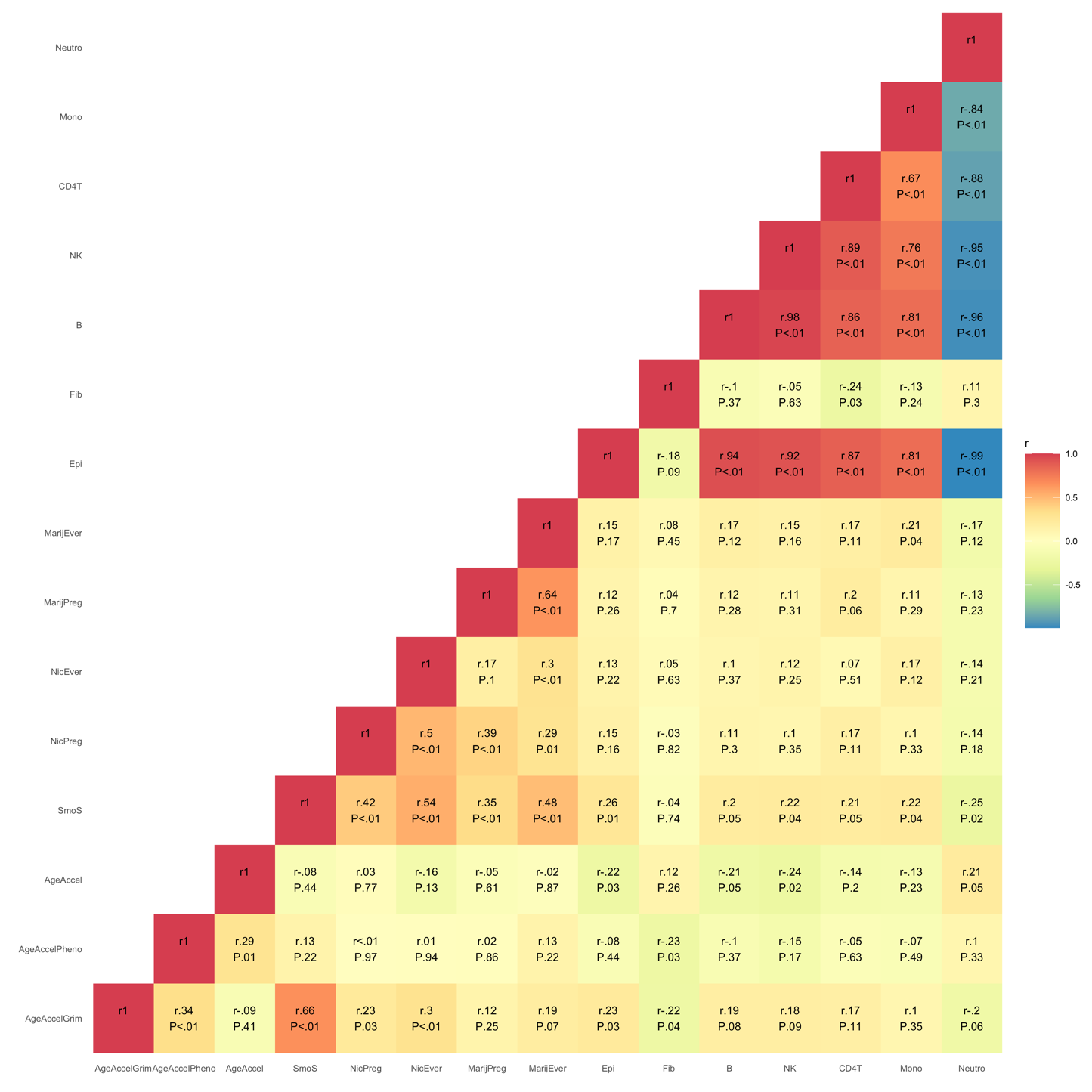


Supplementary Figure 3. Heatmap of correlations between epigenetic age acceleration estimates and study variables. The deeper color indicates stronger correlations. AgeAccelGrim: GrimAge acceleration. AgeAccelPheno: PhenoAge acceleration. AgeAccel: Horvath’s DNAm age acceleration. SmoS: DNAm-based smoking score. NicPreg: Nicotine use during pregnancy. NicEver: Lifetime nicotine use. MarjPreg: Marijuana use during pregnancy. MarjEver: Lifetime marijuana use. Epi: Epithelial cell proportions. Fib: Firbroblast proportions. B: B-cell proportions. NK: Natural killer cell proportions. CD4T: CD4T cell proportions. Mono: Monocyte proportions. Neutro: Neutrophil proportions.


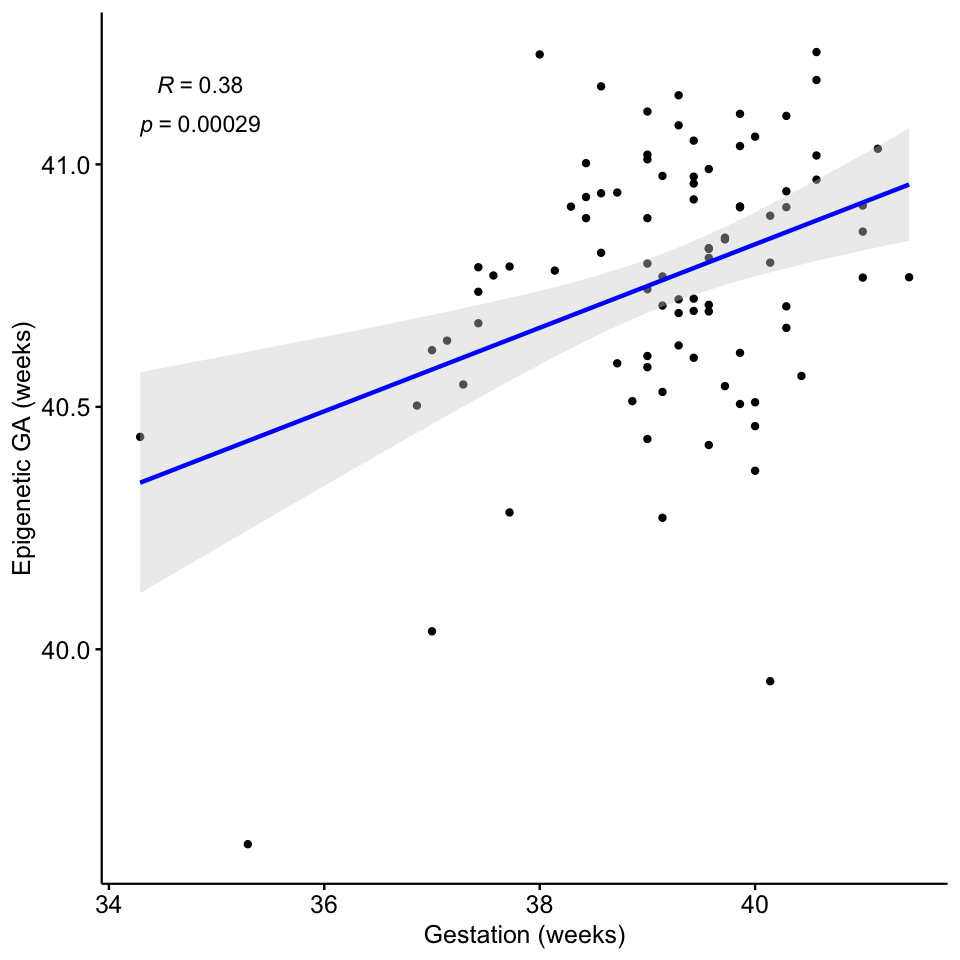


Supplementary Figure 4. Gestational epigenetic age correlates with chronological gestational age of infants. Gray shaded area represents 95% confidence region.


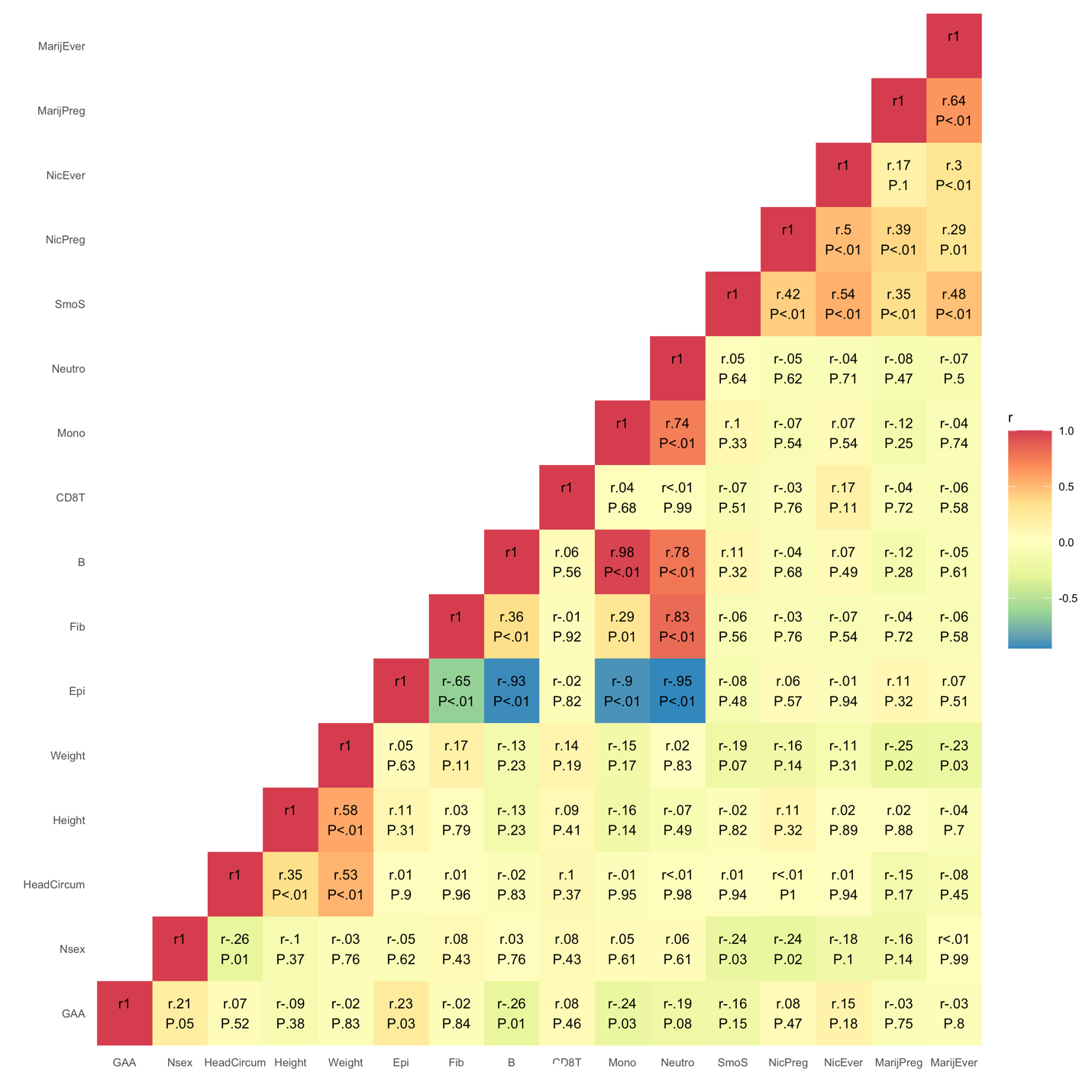


Supplementary Figure 5. Heatmap of correlations between gestational age acceleration and study variables. The deeper color indicates stronger correlations. GAA: Gestational epigenetic age acceleration. Nsex: Neonate sex. HeadCircum: Head circumference. Epi: Epithelial cell proportions. Fib: Firbroblast proportions. B: B-cell proportions. NK: Natural killer cell proportions. CD4T: CD4T cell proportions. Mono: Monocyte proportions. Neutro: Neutrophil proportions.
